# Generative Artificial Intelligence for Chest Radiograph Interpretation in the Emergency Department

**DOI:** 10.1001/jamanetworkopen.2023.36100

**Published:** 2023-10-05

**Authors:** Jonathan Huang, Luke Neill, Matthew Wittbrodt, David Melnick, Matthew Klug, Michael Thompson, John Bailitz, Timothy Loftus, Sanjeev Malik, Amit Phull, Victoria Weston, J. Alex Heller, Mozziyar Etemadi

**Affiliations:** 1 Department of Emergency Medicine, Northwestern University Feinberg School of Medicine, Chicago, Illinois; 2 Research & Development, Northwestern Medicine Information Services, Chicago, Illinois; 3 Department of Anesthesiology, Northwestern University Feinberg School of Medicine, Chicago, Illinois; 4 Department of Biomedical Engineering, McCormick School of Engineering, Northwestern University, Evanston, Illinois

## Abstract

**Importance:**

Multimodal generative artificial intelligence (AI) methodologies have the potential to optimize emergency department care by producing draft radiology reports from input images.

**Objective:**

To evaluate the accuracy and quality of AI–generated chest radiograph interpretations in the emergency department setting.

**Design, Setting, and Participants:**

This was a retrospective diagnostic study of 500 randomly sampled emergency department encounters at a tertiary care institution including chest radiographs interpreted by both a teleradiology service and on-site attending radiologist from January 2022 to January 2023. An AI interpretation was generated for each radiograph. The 3 radiograph interpretations were each rated in duplicate by 6 emergency department physicians using a 5-point Likert scale.

**Main Outcomes and Measures:**

The primary outcome was any difference in Likert scores between radiologist, AI, and teleradiology reports, using a cumulative link mixed model. Secondary analyses compared the probability of each report type containing no clinically significant discrepancy with further stratification by finding presence, using a logistic mixed-effects model. Physician comments on discrepancies were recorded.

**Results:**

A total of 500 ED studies were included from 500 unique patients with a mean (SD) age of 53.3 (21.6) years; 282 patients (56.4%) were female. There was a significant association of report type with ratings, with post hoc tests revealing significantly greater scores for AI (mean [SE] score, 3.22 [0.34]; *P* < .001) and radiologist (mean [SE] score, 3.34 [0.34]; *P* < .001) reports compared with teleradiology (mean [SE] score, 2.74 [0.34]) reports. AI and radiologist reports were not significantly different. On secondary analysis, there was no difference in the probability of no clinically significant discrepancy between the 3 report types. Further stratification of reports by presence of cardiomegaly, pulmonary edema, pleural effusion, infiltrate, pneumothorax, and support devices also yielded no difference in the probability of containing no clinically significant discrepancy between the report types.

**Conclusions and Relevance:**

In a representative sample of emergency department chest radiographs, results suggest that the generative AI model produced reports of similar clinical accuracy and textual quality to radiologist reports while providing higher textual quality than teleradiologist reports. Implementation of the model in the clinical workflow could enable timely alerts to life-threatening pathology while aiding imaging interpretation and documentation.

## Key Points

**Question:**

How do emergency department physicians rate artificial intelligence (AI)–generated chest radiograph reports for quality and accuracy, compared with in-house radiologist and teleradiology reports?

**Findings:**

In this diagnostic study of the developed generative AI model on a representative sample of 500 emergency department chest radiographs from 500 unique patients, the AI model produced reports of similar clinical accuracy and textual quality to radiology reports while providing higher textual quality than teleradiology reports.

**Meaning:**

Results suggest that use of the generative AI tool may facilitate timely interpretation of chest radiography by emergency department physicians.

## Teaser

This diagnostic study investigates the quality and accuracy of artificial intelligence–generated chest radiography reports compared with the reports of in-house radiologists and teleradiology.

## Introduction

In the emergency department (ED), timely interpretation of diagnostic imaging is a crucial component in clinical decision-making for otherwise undifferentiated patients. Although ED physicians interpret chest radiographs with rates of clinically significant discrepancy from 1% to 2% compared with radiologists,^1,2^ immediate radiologist interpretation may further minimize treatment-altering differences and reduce callbacks of patients discharged from the ED.^2,3^ In light of rising imaging utilization in the ED,^4^ systems for providing prompt interpretation have become increasingly important to streamline emergency care.

However, free-standing EDs may lack dedicated radiology services and centers may not provide off-hours coverage. This gap is typically filled by preliminary resident interpretations or teleradiology services,^5^ solutions made less than ideal given the potential for discrepant reporting by trainees or outside radiologists without access to the full clinical record.^6,7^ Discrepancies found when preliminary reads are overread by an on-site radiologist may necessitate further intervention, despite the patient having been discharged. Thus, improvement of ED physician access to radiology services in lower-resourced settings is desirable.

Generative artificial intelligence (AI) methods, which generate data such as text and images following user direction,^8^ may bridge this gap by providing near-instant interpretations of medical imaging, supporting high case volumes without fatigue or personnel limitations. An important advantage of the generative approach over classification methods is the ability to produce more informative and relevant outputs via generation of the entire radiology report, providing important context for decision-making in the ED. However, clinically oriented evaluations of generative AI remain scarce in the biomedical literature.^9^ Considering the importance of both qualitative and quantitative components to radiology report quality, evaluation by potential physician end users is needed to assess the clinical utility of AI-generated radiograph reports. This is particularly relevant in the ED, where physicians rely on imaging interpretations to provide clear reporting on findings requiring immediate intervention. The aim of this study was to develop a generative AI tool for chest radiograph interpretation and retrospectively evaluate its performance in the ED setting.

## Methods

The protocol for this diagnostic study was approved by the Northwestern University institutional review board. A waiver of participant consent was granted. Study reporting followed the Standards for Reporting of Diagnostic Accuracy (STARD) reporting guidelines.

### AI Model Architecture

**Figure 1.**
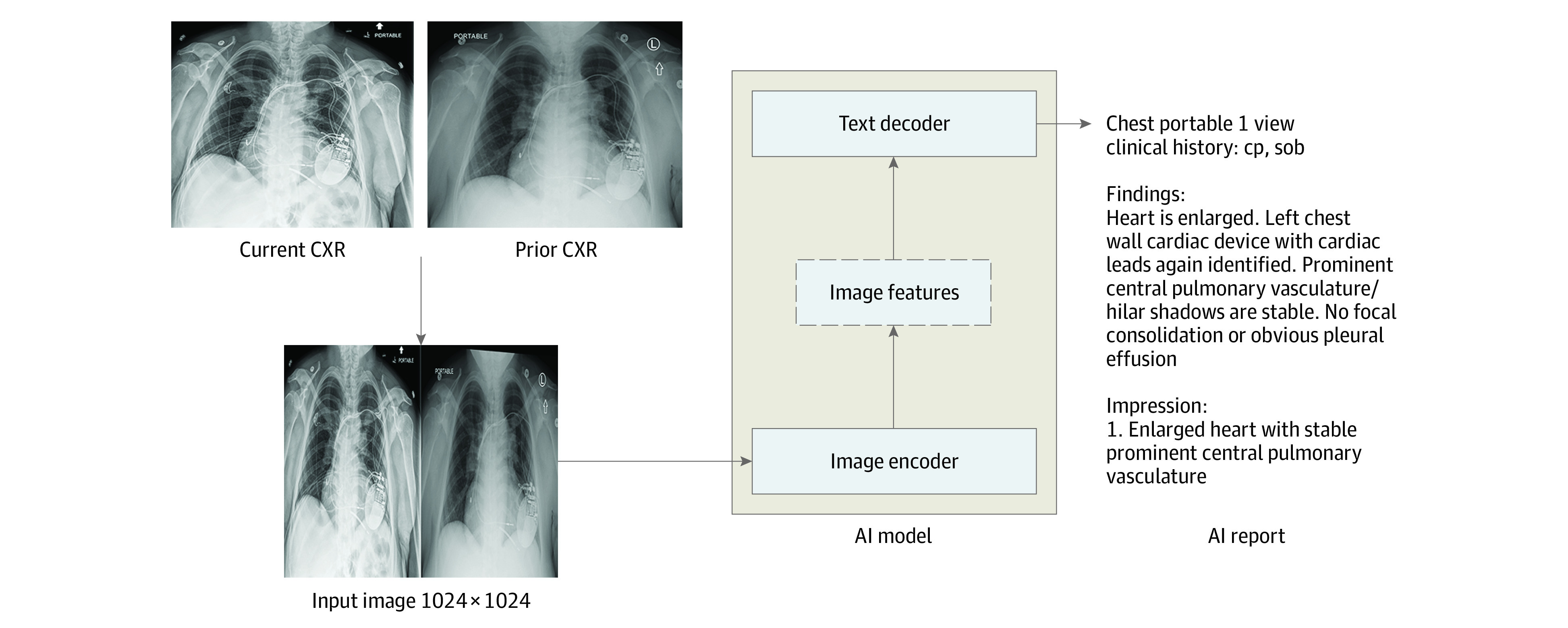
Artificial Intelligence (AI) Model Architecture The AI model is an encoder-decoder model trained to generate a text report given a chest radiograph (CXR) and most recent comparison (anterior-posterior or posterior-anterior view only). The vision encoder weights were initialized from Vision Transformer (ViT) base and the text decoder weights were initialized from Robustly Optimized BERT Pretraining Approach (RoBERTa) base before training for 30 epochs on a data set of 900 000 CXRs. cp indicates chest pain; sob, shortness of breath.

Briefly, the AI tool is a transformer-based encoder-decoder model that takes chest radiograph images as input and generates radiology report text as output (Figure 1). Model architecture and development details are provided in the eMethods in Supplement 1.

### ED Test Data Set

**Figure 2.**
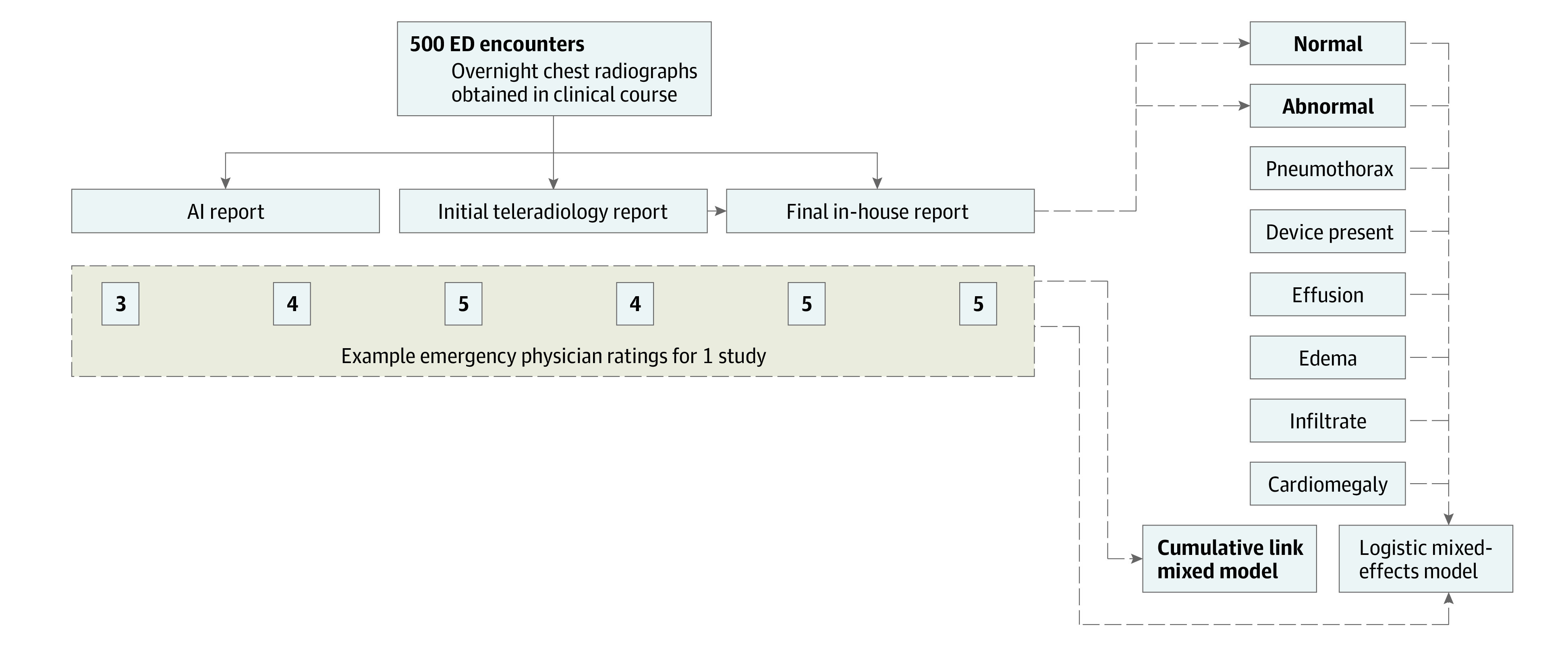
Artificial Intelligence (AI) Evaluation Study Design A total of 500 emergency department (ED) encounters with associated overnight chest radiographs interpreted by a teleradiology service, then overread by an in-house radiologist, were randomly selected. The teleradiology reports as well as the finalized in-house radiologist reports were retrospectively identified, and an AI report was generated as well. Six ED physicians served as raters; each report was rated for accuracy and quality by 2 physicians blinded to the report type using a 5-point Likert scale such that each physician rated each chest radiograph once. The primary and secondary analyses were also performed as shown.

The test data set comprised 500 randomly sampled anterior-posterior (AP) or posterior-anterior (PA) chest radiographs of patients evaluated in an ED at our institution, for which both a teleradiology and final radiologist report were available (Figure 2). Teleradiology is routinely consulted for all overnight imaging at institutional EDs without overnight in-house radiology coverage. Radiologist reports overreading teleradiology are documented by an attending physician without resident input. Sampling was limited to encounters from January 2022 to January 2023 and excluded patients in the model development data set. Patients younger than 18 years or older than 89 years were excluded. Participant race and ethnicity were not gathered for this study, as this information is not relevant to chest radiograph interpretation and is not routinely available to radiologists. The most recent prior AP or PA chest radiographs were also identified, if present, and were used to save model input images. Model inference was performed to generate an AI report for each image, using 4 deterministic beams and typical decoding^10^ with a parameter value of 0.95. Corresponding teleradiology and radiologist reports were obtained, and all 3 reports were deidentified. As institutional practice is for radiologists to comment on their agreement with preliminary reports, all such references were removed.

Study acquisition techniques were extracted from Digital Imaging and Communications in Medicine, or DICOM, files. Comparison intervals were expressed as same day or an interval of days, weeks, months, or years before the current image. Clinical indications were extracted from the radiologist report. Finally, reports were truncated to include only the Findings and Impression/Conclusion(s) sections or other interpretation text.

### ED Physician Ratings

A total of 6 practicing board-certified emergency medicine physicians (L.N., J.B., T.L., S.M., A.P., and V.W.) served as raters (Figure 2). Raters used a custom webpage to rate reports; each page displayed the original resolution current and prior chest radiograph images, the acquisition type along with comparison interval and indication, and the report body randomly selected from the 3 report types. Each physician rated all 500 studies exactly once in an individually randomized order, with assignment such that each study received 2 ratings per report type.

**Figure 3.**
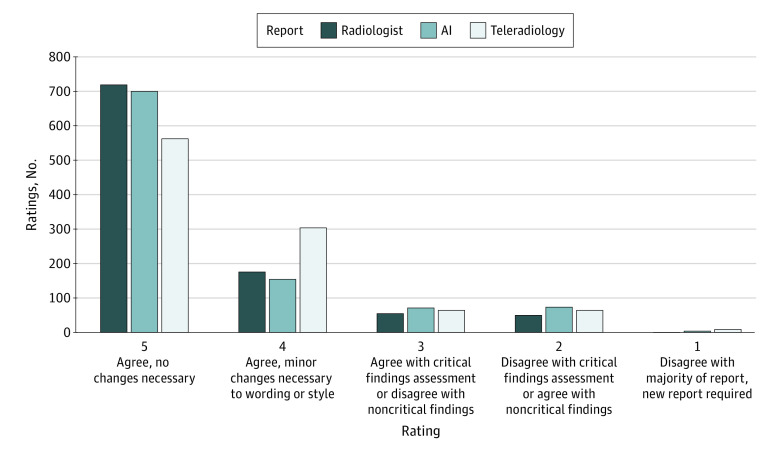
Overall Rating Distribution The distribution of Likert scale ratings for radiologist, artificial intelligence (AI), and teleradiology reports is shown. Each report was rated in duplicate, resulting in 1000 ratings of 500 radiographs for each of the 3 report types.

A Likert scale (Figure 3) was used to rate report quality and clinical accuracy. A critical finding was defined to be one that would change the physician’s clinical management of the patient in the ED if reported incorrectly. Raters were instructed to use a comments text field on each page to describe any discrepancies for studies rated 3 or lower (ie, if the study missed any finding).

### Statistical Analysis

Likert scores between radiologist, AI, and teleradiology reports were compared using a cumulative link mixed model from the ordinal (version 2022.11-16) package in R (version 4.3.0 [R Project for Statistical Computing]) fit with main effects of report type and whether a finding was found in the original report, along with the interaction effect and random effects of patient and rater. A secondary analysis was completed by binarizing Likert scores by presence (rating <3) or absence (rating ≥3) of a clinically significant discrepancy. A generalized logistic mixed-effects model was fit using the same parameters. Furthermore, analysis of clinically significant discrepancies was conducted on subgroups using a main effect of report type and random effect of patient to investigate whether clinically significant reporting errors were made in studies with findings of cardiomegaly, edema, effusion, infiltrate, pneumothorax, or support device presence. Model results are reported as estimated marginal means and SE, unless otherwise noted. For all analyses, if a significant main effect was found, post hoc analyses were completed with the emmeans (version 1.8.6) package in R with Tukey corrections. To examine the within-report-type rating concordance, Kendall *W* was calculated for each report type using DescTools (version 0.99.49) in R using corrections for tied rankings. Finally, all reports were categorized as abnormal or normal using the radiologist report as a criterion standard, and sensitivity and specificity of AI and teleradiology reports for detection of abnormality were calculated. The α level was set to *P* ≤ .05 to determine significance, and all *P* values were 2-sided.

## Results

The test data set contained 500 ED studies from 500 unique patients, of whom 282 (56.4%) were female and 218 (43.6%) were male. Patients had a mean (SD) age of 53.3 (21.6) years. There were 336 normal radiographs (67.2%) and 164 abnormal radiographs (32.8%). A total of 434 radiographs (86.8%) were portable AP acquisitions, with 65 PA and lateral (13.0%) and 1 upright PA film (0.2%). The most common findings were infiltrates (71 [14.2%]), pulmonary edema (47 [9.4%]), pleural effusions (43 [8.6%]), support device presence (37 [7.4%]), cardiomegaly (21 [4.2%]), and pneumothorax (4 [0.8%]). The 6 ED physician raters had a mean (SD) of 10.5 (6.4) years of postresidency clinical practice experience. The 12 on-site, board-certified diagnostic radiologists who interpreted the ED studies had a mean (SD) of 14.6 (12.5) years of postresidency clinical practice experience. All teleradiologists completed both residency and board certification in the US. Examples of reports, ratings, and comments containing discrepancies between report types are given in the eTable in Supplement 1.

The overall distribution of assigned ratings is shown in Figure 3. Kendall *W* values were 0.536, 0.526, and 0.512 for radiology, AI, and teleradiology reports, respectively, indicating moderate interrater agreement within each report type. Examining raw Likert scores, the main effect of finding (no finding: mean [SE], 3.23 [0.48]; finding: mean [SE], 2.98 [0.25];* P = *.51) and interaction between finding and report type were not significant. However, scores did differ significantly based on the main effect of report type, with post hoc tests revealing significantly greater ratings for AI (mean [SE], 3.22 [0.34];* P < *.001) and radiologist (mean [SE], 3.34 [0.34];* P < *.001) reports compared with teleradiology (2.74 [0.34]) reports. Ratings of AI and radiologist reports were not significantly different.

**Figure 4.**
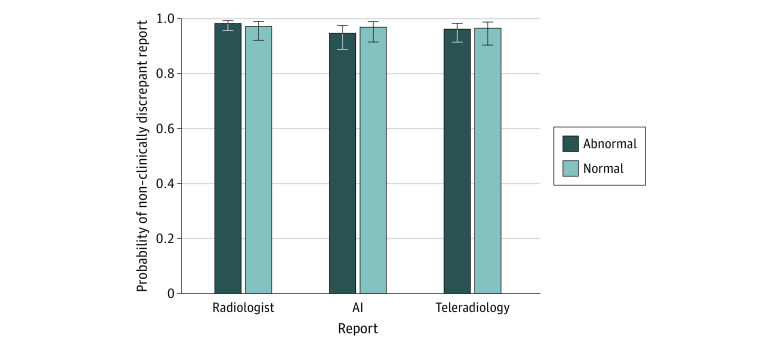
Probability of Non–Clinically Discrepant Report The probability of producing a non–clinically discrepant report (ie, Likert score ≥3) for studies with and without an abnormality across each report type. Error bars designate the upper and lower confidence limits of the probability estimate. AI indicates artificial intelligence.

**Figure 5.**
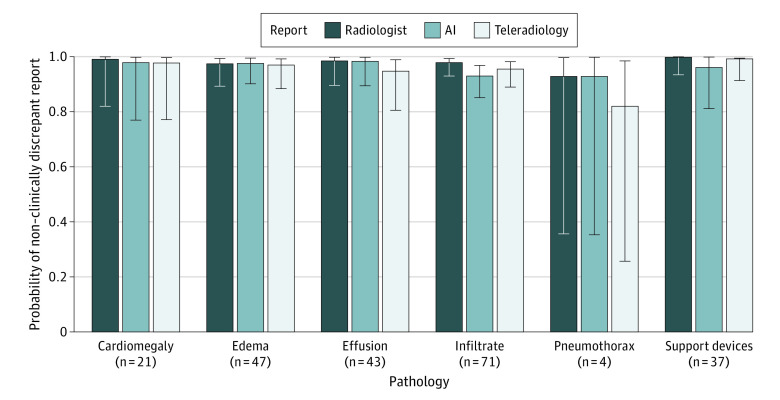
Probability of Non–Clinically Discrepant Report Across Pathologies The probability of producing a non–clinically discrepant report (ie, Likert score ≥3) for each read type across subsets of studies with a given abnormality. Error bars designate the upper and lower confidence limits of the probability estimate. The number below each label indicates the study count for that subset. AI indicates artificial intelligence.

Figure 4 presents the probability that each report type would generate a non–clinically discrepant report for normal and abnormal studies. The analysis of clinical significance resulted in no significant main effect of report type (radiologist: mean [SE], 0.98 [0.01]; AI: mean [SE], 0.96 [0.01]; teleradiology: mean [SE], 0.94 [0.02];* P = *.12) or finding (finding: mean [SE], 0.97 [0.01]; no finding: mean [SE], 0.97 [0.01];* P = *.64); the report type by finding interaction was also not significant. Figure 5 presents the probability of each report type generating a non–clinically discrepant report for subsets of data by finding presence. There were also no significant main effects of report type for studies featuring cardiomegaly, pulmonary edema, pleural effusion, infiltrate, pneumothorax, and support devices.

Each report rated less than 3 on the Likert scale, which indicated a change in clinical management, was reviewed, and the discrepant finding categorized as missed (a significant finding was not commented on), extraneous (a finding commented on was not deemed present), or improperly contextualized (a finding was presented without proper contextual information, eg, degree of severity or change from prior misstated), based on the rater comment. Of 51 discrepant radiologist report findings, 36 (70.6%) were commented on; 33 (91.7%) were missed, 2 (5.5%) were extraneous, and 1 (2.8%) was improperly contextualized. Of 76 discrepant AI report findings, 52 (68.4%) were commented on; 42 (80.8%) were missed, 1 (1.9%) was extraneous, and 9 (17.3%) were improperly contextualized. Lastly, of 72 discrepant teleradiology report findings, 46 (63.9%) were commented on; 39 (84.8%) were missed, 2 (4.3%) were extraneous, and 5 (10.9%) were improperly contextualized.

Finally, using radiologist reports as the criterion standard, the sensitivity and specificity of AI reports for detecting any abnormality were 84.8% and 98.5%, respectively. Teleradiology reports had a sensitivity of 91.5% and specificity of 97.0% for the same task.

## Discussion

As the potential for generative AI methods to accelerate clinical decision-making and documentation continues to grow, demonstration of efficacy in real-world clinical settings is paramount. Given the challenge of objectively evaluating the accuracy of free-text imaging interpretations,^11^ input from ED physician end users is needed to assess qualities of the AI report salient to their particular practice setting. We introduced and retrospectively evaluated a generative AI tool for chest radiograph report generation in the ED setting, using Likert scale ratings to evaluate report accuracy and textual quality in relation to potential impacts to ED physician decision-making. AI-generated reports were not significantly different from radiologist reports, although they performed better than teleradiology reports, providing evidence for the applicability of AI to supplement ED physician decision-making in settings without immediate access to radiology services.

To our knowledge, this was the first study to evaluate the report generation approach to chest radiograph interpretation by an AI language model in a clinical setting. Studies published to date have primarily used classification approaches, predicting the presence of individual pathologies.^12,13,14,15^ However, reducing medical diagnoses to binary predictions of presence or absence may omit context relevant to clinical care, such as the severity, location, and clinical course of a finding. For instance, the presence of a pneumothorax has very different implications in a newly presenting patient as opposed to a patient with pneumothorax improvement after chest tube placement. As our AI tool generates radiologist-style report text using both the current and most recent prior radiographs, it can contextualize findings where a classification-based model would not, even providing differential diagnoses and recommendations for further evaluation.

The utility of this additional context was evident in significantly higher ED physician ratings of AI compared with teleradiology reports, with an overall small effect (estimated mean difference: 0.49) likely attributable to a larger percentage of teleradiology reports rated a 4 rather than 5. Teleradiology reports tended to be more terse and less structured than the other report types, only reporting the word “normal” in the extreme case (eTable in Supplement 1). Physicians commonly noted that teleradiology reports omitted mention of support devices or pertinent negative findings. As the AI model was trained on institutional data, the generated reports follow institutionally standardized structured formatting, which enforces reporting on relevant aspects of the chest radiograph even in the absence of abnormality. Structured reports may more effectively convey relevant information to ED physicians, highlighting the importance of AI models institutionally tailored to fit the needs of their own patient population and clinicians.

In the present study, interrater reliability was moderate between pairs of ratings for all 3 report types, suggesting that there is genuine clinical uncertainty in many radiographic findings. Studies have shown relatively low interreader reliability on chest radiography interpretation across many different pathologies,^16,17,18^ although double reading of chest radiographs increases sensitivity for pathology.^19^ For context, abnormal radiographs are frequently misdiagnosed by trainees, with accuracy as low as 9% for conditions such as pneumothorax.^20,21^ Among radiologists, studies have demonstrated a discrepancy rate of 4% in representative samples of chest radiographs, rising to as high as 30% when abnormal radiographs are exclusively considered.^22^ The low rates of clinically significant discrepancy in AI reports in studies containing actionable findings—nearly one-third of the test data set—compares favorably to these benchmarks, highlighting the relevance of AI reports to ED physicians.

Notably, there were several cases in which the AI report improved on the radiologist report. For instance, in 1 case, both raters commented that the radiologist report missed a new infiltrate that was correctly described in the AI report. In another, the radiologist report described opacities as “persistent” compared with the prior image, whereas the AI and teleradiology reports noted that this had worsened, in agreement with raters (eTable in Supplement 1). As ED physicians must maintain high sensitivity for clinically significant findings when reviewing imaging, use of this AI tool could call to attention potentially overlooked abnormalities, serving in a preliminary capacity similar to teleradiologist interpretations in ED workflows.

As AI reports can be generated within seconds of radiograph acquisition, real-time review could notify physicians of potential abnormalities, aiding in triage and flagging critical findings requiring early intervention. The results of the current study suggest that the AI model was similarly proficient in identifying these clinical abnormalities as a radiologist. Notably, the AI report correctly identified all 4 pneumothoraces in the test data set. Several automated tools in clinical use screen radiology reports for relevant findings and trigger appropriate clinical workflows in response,^23,24^ the timeliness of which could be increased with integration of our model. With a specificity of 98.5% for abnormal findings, studies could be prioritized with high confidence.

Although the main effect was not significant, physician feedback has indicated that AI reports for studies containing multiple support devices presented difficulty for the model. Occasional inconsistent reporting of numeric values was noted throughout development and evaluation, which particularly impacted reporting of endotracheal tube positioning relative to the carina. The greater proportion of contextual errors among the AI report discrepancies reflects this finding. Importantly, the training data contained no indication of pixel scale, which is available to radiologists. Regardless, difficulties of generative language models with numerical reasoning have been well documented,^25,26^ with proposed solutions such as chain-of-thought reasoning^27^ and verification of outputs by separate models.^26^ Methods such as reinforcement learning with human feedback^28^ offer another potential avenue for improving clinical accuracy and steering language models to the varying needs of different clinical domains. These results also highlight the importance of keeping a “human in the loop” when using AI for clinical care.

There are famously many limitations to language models and AI tools in general. In 2016, a prominent AI expert claimed that in 5 years, there would no longer be any need for human radiologists,^29^ sparking intense discussion on the role of AI in the interpretation of medical imaging. Clearly, this has not been the case; as limitations of generative models in nonmedical contexts continue to emerge, efforts are needed to mitigate potential effects in the clinical setting. Regardless, there is a clear opportunity for generative AI to augment clinical care. Ultimately, we believe that this study highlights the value of collaborative physician-AI synergy, demonstrating a promising application of generative AI to complement physician decision-making in real clinical settings.

### Limitations

Some limitations warrant consideration. As only the ED setting was studied, model generalizability to other settings and institutions remains unclear. Moreover, less common pathologies such as mediastinal widening were not well represented. Additionally, as studies were categorized as normal or abnormal based on the radiologist report as the ground truth, inaccuracies by the reading radiologist may have affected our analysis of rating distributions stratified by pathology.

Another limitation is that radiologist reports overread preliminary teleradiology reports and thus received input from 2 separate radiologists. Considering the known benefit of double reading on the sensitivity of radiologist reporting,^19^ it is plausible that this study underestimates AI performance relative to an independent on-site radiologist. This may also have led to an underestimation of the sensitivity and specificity of the AI tool for abnormality detection.

Accuracy of free-text chest radiograph reports remains notably difficult to quantify,^11^ particularly considering interrater variability inherent in chest radiograph interpretation as well as differing practice patterns among physician raters. The current study performed ratings in duplicate to mitigate and understand this effect; however, further work is needed, such as evaluation by radiologists to assess suitability in a general radiology practice setting.

Finally, as the model was trained using only AP and PA chest radiographs of lower resolution (approximately 1 megapixel) than those available to radiologists (approximately 4 megapixels), there is potentially a loss of information in the lateral view or at higher resolutions. A relatively small proportion of evaluation studies included a lateral view, and no significant difference in ratings was found between studies with and without a lateral view, suggesting that the AI tool can nonetheless generate reports of sufficient quality. Effective integration of clinical information into AI models requires further study.

## Conclusions

In this diagnostic study accounting for both clinical accuracy and textual quality, results suggest that our AI tool produced reports similar in performance to a radiologist and better than a teleradiology service in a representative sample of ED chest radiographs. AI report ratings were comparable with those of on-site radiologists across all evaluated pathology categories. Model integration in clinical workflows could enable timely alerts to life-threatening pathology while aiding physician imaging interpretation and speeding up documentation. Further efforts to prospectively evaluate clinical impact and generalizability are needed.

## Notes

Supplement 1. **eMethods.** AI Model Development **eTable.** Example Discrepant Reports With Emergency Physician Ratings and Comments
eReferences

Supplement 2.
Data Sharing Statement
