# Supplementary material for: Generative Artificial Intelligence for Chest Radiograph Interpretation in the Emergency Department
Source: JAMA Netw Open. 2023 Oct 5;6(10):e2336100. doi: 10.1001/jamanetworkopen.2023.36100 (PMC10556963; doi:10.1001/jamanetworkopen.2023.36100)
Supplement: Supplement 1. — eMethods. AI Model Development eTable. Example Discrepant Reports With Emergency Physician Ratings and Comments eReferences [file jamanetwopen-e2336100-s001.pdf]

# Supplementary Online Content

Huang J, Neill L, Wittbrodt M, et al. Generative artificial intelligence for chest radiograph interpretation in the emergency department. *JAMA Netw Open*. 2023;6(10):e2336100. doi:10.1001/jamanetworkopen.2023.36100

**eMethods.** AI Model Development

**eTable.** Example Discrepant Reports With Emergency Physician Ratings and Comments

**eReferences**

This supplementary material has been provided by the authors to give readers additional information about their work.

© 2023 Huang J et al. *JAMA Network Open.*

## eMethods. AI Model Development

### *AI Model Architecture*

The vision encoder portion is a base-sized vision transformer model with 12 layers, 12 attention heads, hidden size 768, and patch size of 16 pre-trained on ImageNet-21k at resolution 224x224 and fine-tuned on ImageNet2012 at resolution 384x384.[1] Encoder weights were initialized from https://huggingface.co/google/vit-base-patch16-384. The text decoder portion is a pretrained base-sized RoBERTa model (initialized in decoder mode) with 12 layers, 12 attention heads, and hidden size 768 pre-trained on BookCorpus and English Wikipedia.[2] Decoder weights were initialized from https://huggingface.co/roberta-base. The entire model contained 210 million parameters.

### *Model Training Dataset*

The AI model training dataset comprised anteroposterior (AP) and posteroanterior (PA) chest radiographs obtained between January 2020 and December 2021 at our institution. Additionally, the most recent prior AP or PA chest radiograph for each study was also included if present in the institutional database. All images were square-padded by adding black bars in either the vertical or horizontal directions, maintaining the image in the center. Images were then resized to size 1024 x 1024 using bilinear interpolation and normalized to mean 0.5 and standard deviation 0.5, then replicated across all three RGB image channels. Studies without a prior comparison were then saved. For studies with a prior comparison, the current and prior images were concatenated with the current image on the left and the prior image on the right, and the resulting image resized to 1024 x 1024 (**Figure 1**). Radiology reports were tokenized using the base RoBERTa tokenizer.

The train and validation split was performed at the patient level. 269,028 patients, comprising 900,000 unique images, served as the training set. The remaining 41,264 patients and 108,658 images served as the validation set.

### *Model Training Method*

The AI model was trained for 30 epochs on 8 NVIDIA (Santa Clara, CA) A100 40GB graphics processing units (GPUs) with a global batch size of 80 (microbatch 1 per device, accumulation of 10) and fixed learning rate of 0.00005. Image position encodings were interpolated in order to fine tune the vision encoder at the full input resolution (1024x1024). The total training time was approximately 18 days.

© 2023 Huang J et al. *JAMA Network Open.*

**eTable. Example Discrepant Reports With Emergency Physician Ratings and Comments**

| Radiologist report | AI report | Teleradiology report |
|---|---|---|
| **Report text:**<br><br>FINDINGS:<br>No enlargement of the cardiomediastinal silhouette.. The lungs are clear without infiltrates.<br><br>IMPRESSION:<br>No acute intrathoracic findings.<br><br>**Ratings:**<br><br>5, 5<br><br>**Comments:**<br><br>n/a | **Report text:**<br><br>FINDINGS:<br>The lungs are clear. The heart size is normal. The mediastinal contour is within normal limits. There is no pulmonary consolidation, pleural effusion or mass lesion seen. The bony structures are grossly intact.<br><br>IMPRESSION:<br>No acute disease.<br><br>**Ratings:**<br><br>5, 5<br><br>**Comments:**<br><br>n/a | **Report text:**<br><br>Normal<br><br>**Ratings:**<br><br>5, 1<br><br>**Comments:**<br><br>n/a |
| **Report text:**<br><br>FINDINGS:<br>There is a poor inspiratory effort. Bilateral pleural effusions are present. There are scattered bilateral parenchymal opacities. Cardiomegaly with pulmonary vascular congestion is also present. There is no pneumothorax. There is no change in the right-sided Port-A-Cath.<br><br>IMPRESSION: 1. Persistent bilateral parenchymal opacities and pleural effusions.<br><br>**Ratings:**<br><br>4, 2 | **Report text:**<br><br>FINDINGS:<br>Right-sided chest port placement is noted.<br>Ill-defined opacities are demonstrated in the bilateral mid to lower lung zones, compatible with acute airspace disease. It has worsened since prior study.<br>There is blunting of the bilateral costophrenic angles, compatible with small bilateral pleural effusions, left greater than right.<br>The heart size is normal. The mediastinal contour is within normal limits.<br><br>IMPRESSION:<br>1. Bilateral acute airspace disease, worsened since prior study. | **Report text:**<br><br>Cardiomegaly.<br><br>Bilateral increased reticular interstitial opacities consistent with progression of interstitial pneumonia.<br>Bilateral small pleural effusions.<br><br>Right-sided portacatheter tip at the superior vena cava.<br><br>**Ratings:**<br><br>5, 5<br><br>**Comments:** |

© 2023 Huang J et al. *JAMA Network Open.*

| Radiologist report | AI report | Teleradiology report |
|---|---|---|
| **Comments:**<br><br>Increase in R sided pleural effusion, stable L sided. Bilateral opacities.<br><br>Significant worsening | 2. Small bilateral pleural effusions, left greater than right.<br><br>**Ratings:**<br><br>5, 4<br><br>**Comments:**<br><br>n/a | n/a |
| **Report text:**<br><br>FINDINGS:<br>Bilateral chest tube placement with decreased bilateral pneumothoraces and a trace. Enteric tube into the stomach and endotracheal tube 4 cm above carina. Bilateral peripheral predominant opacities similar to prior accounting for differences in lung expansion. Diffuse subcutaneous emphysema as before.<br><br>IMPRESSION:<br>Placement of bilateral chest tubes with decrease in pneumothoraces.<br><br>**Ratings:**<br><br>4, 4<br><br>**Comments:**<br><br>n/a | **Report text:**<br><br>FINDINGS:<br>The tip of the ET tube is about 4.5 cm above the carina. The patient is status post NG tube placement with its tip projecting over the fundus of the stomach.<br>Small pigtail chest tube placement is noted on the right side, new since prior study. The tip of a right-sided pigtail chest tube placement is projecting over the lateral mid hemithorax.<br>Small bilateral pneumothoraces are demonstrated, decreased on the right side and stable on the left side when compartment prior study.<br>Extensive soft tissue emphysema is noted along the bilateral lateral chest walls and the visualized upper abdomen.<br>Diffuse patchy opacities are noted in both lungs, stable since prior study. The heart size is normal. The mediastinal contour is within normal limits.<br><br>IMPRESSION:<br>1. Patient is status post ET, NG and right pigtail chest tube placement.<br>2. Small bilateral pneumothoraces, | **Report text:**<br><br>Interval placement of bilateral chest tubes. Interval decrease in size of bilateral pneumothoraces, now both trace.<br><br>ET tube terminates approximately 3.8 cm above the carina.<br>Persistent cutaneous emphysema throughout the chest and supraclavicular soft tissues.<br>An enteric tube terminates in the stomach.<br><br>Persistent multifocal opacities throughout the left lung and interstitial opacities throughout the right lung.<br><br>**Ratings:**<br><br>5, 5<br><br>**Comments:**<br><br>n/a |

© 2023 Huang J et al. *JAMA Network Open.*

| Radiologist report | AI report | Teleradiology report |
| --- | --- | --- |
| | decreased on the right side and stable on the left side when compared to prior study.<br>3. Otherwise stable findings since the prior study.<br><br>**Ratings:**<br><br>5, 3<br><br>**Comments:**<br><br>Left side chest tube | |
| **Report text:**<br><br>FINDINGS:<br>Redemonstrated are sternotomy wire and mediastinal postoperative changes. The heart is not enlarged. The pulmonary vessels are normally distributed. There is calcific plaque along the aortic knob. The lungs are clear The costophrenic angles are sharp. The mediastinum is not widened. There is no pneumothorax. No acute bony abnormality is evident.<br><br>IMPRESSION:<br>1. No acute cardiopulmonary findings.<br><br>**Ratings:**<br><br>5, 5<br><br>**Comments:**<br><br>n/a | **Report text:**<br><br>FINDINGS:<br>There are multiple midline sternotomy wires noted.<br>The lungs are clear. The heart size is normal. The mediastinal contour is within normal limits. There is no pulmonary consolidation, pleural effusion or mass lesion seen. The bony structures are grossly intact.<br><br>IMPRESSION: No acute disease.<br><br>**Ratings:**<br><br>5, 4<br><br>**Comments:**<br><br>n/a | **Report text:**<br><br>No acute cardiopulmonary abnormality.<br><br>**Ratings:**<br><br>3, 2<br><br>**Comments:**<br><br>Appearance of worsening pulmonary edema. Sternal wires noted. |
| **Report text:** | **Report text:** | **Report text:** |

© 2023 Huang J et al. *JAMA Network Open.*

| Radiologist report | AI report | Teleradiology report |
|---|---|---|
| FINDINGS:<br>Limitation by portable technique.  Top normal heart size.  Moderate elevation of right hemidiaphragm with right basilar opacities.  Prominent right infrahilar shadow adjacent to elevated diaphragm.  Linear opacities in left lower lung.  No obvious pleural effusion.<br><br>IMPRESSION:<br>1.  Moderate elevation of right hemidiaphragm with right basilar opacities and prominent right infrahilar shadow.  Cannot exclude infiltrates or other etiologies.  Recommend radiographic follow-up.<br><br>**Ratings:**<br><br>5, 4<br><br>**Comments:**<br><br>n/a | FINDINGS:<br>There is moderate elevation of the right hemidiaphragm.  Linear densities are seen in the right lung base, suggesting subsegmental atelectasis.<br>The left lung is clear.  The heart size is normal.  The mediastinal contour is within normal limits.  No large pleural effusion is demonstrated.<br><br>IMPRESSION: Elevated right hemidiaphragm with right basilar atelectasis.<br><br>**Ratings:**<br><br>5, 4<br><br>**Comments:**<br><br>Atelectasis vs infiltrate RML | Elevated right hemidiaphragm. Prominent right hilar structures abutting the elevated right hemidiaphragm Nonspecific.<br><br>Mild scattered scarring/chronic changes. No focal consolidation. No large effusion.<br><br>**Ratings:**<br><br>5, 3<br><br>**Comments:**<br><br>Possible small R pleural effusion above elevated HD, minimal clinical impact |

## eReferences


1. Dosovitskiy A, Beyer L, Kolesnikov A, et al. An Image is Worth 16x16 Words: Transformers for Image Recognition at Scale. 2021.
2. Liu Y, Ott M, Goyal N, et al. RoBERTa: A Robustly Optimized BERT Pretraining Approach. *CoRR.* 2019;abs/1907.11692.


© 2023 Huang J et al. *JAMA Network Open.*
